# Human *PPP1R26P1* Functions as *cis*-Repressive Element in Mouse *Rb1*

**DOI:** 10.1371/journal.pone.0074159

**Published:** 2013-09-03

**Authors:** Laura Steenpass, Deniz Kanber, Michaela Hiber, Karin Buiting, Bernhard Horsthemke, Dietmar Lohmann

**Affiliations:** Institut für Humangenetik, Universitätsklinikum Essen, Universität Duisburg-Essen, Essen, Germany; Montana State University, United States of America

## Abstract

The human retinoblastoma gene (*RB1*) is imprinted; the mouse *Rb1* gene is not. Imprinted expression of *RB1* is due to differential methylation of a CpG island (CpG85), which is located in the pseudogene *PPP1R26P1* in intron 2 of *RB1*. CpG85 serves as promoter for an alternative *RB1* transcript, which is expressed from the unmethylated paternal allele only and is thought to suppress expression of the full-length *RB1* transcript *in cis*. *PPP1R26P1* contains another CpG island (CpG42), which is biallelically methylated. To determine the influence of *PPP1R26P1* on *RB1* expression, we generated an *in vitro* murine embryonic stem cell model by introducing human *PPP1R26P1* into mouse *Rb1*. Next generation bisulfite sequencing of CpG85 and CpG42 revealed differences in their susceptibility to DNA methylation, gaining methylation at a median level of 4% and 18%, respectively. We showed binding of RNA polymerase II at and transcription from the unmethylated CpG85 in *PPP1R26P1* and observed reduced expression of full-length *Rb1* from the targeted allele. Our results identify human *PPP1R26P1* as a *cis*-repressive element and support a connection between retrotransposition of *PPP1R26P1* into human *RB1* and the reduced expression of *RB1* on the paternal allele.

## Introduction

Biallelic loss-of-function of the *RB1* gene is causal for retinoblastoma, the most common eye tumor in early childhood [1]. This gene was found to be imprinted in humans [2]. The murine ortholog, however, is not imprinted [2,3]. Genomic imprinting manifests in parent-of-origin-dependent gene expression that is controlled by a differentially methylated region (differentially methylated region) *in cis*, which becomes methylated in the germline of only one of the two sexes. Differential methylation of an imprinted gametic differentially methylated region needs to be stably maintained and inherited from cell to cell during development and lifetime of the organism [4,5].

Parent-of-origin dependent expression of the *RB1* gene is linked to a differentially methylated region in intron 2 of *RB1* that is derived from a retrotransposed copy of *PPP1R26* (former name *KIAA0649*) [2]. Retrotransposition is the integration of a reverse-transcribed cellular mRNA into a new genomic location, generating processed pseudogenes, having no function in most cases [6]. If, however, these processed pseudogenes maintain or gain expression of mRNA and protein, they are called retrogenes [7]. Some retrogenes acquire parental-specific differential methylation, turning them into imprinted retrogenes [8]. In the mouse, imprinted retrogenes located in an intron of a host gene, often confer tissue-specific imprinted expression on the host gene itself, like in the ‘intronic-host’ pairs *U2af1-rs1/Commd1, Mcts2*/*H13* and *Nap1l5*/*Herc3* [8–11]. In the human, however, the host genes of the orthologues of these imprinted retrogenes are not imprinted [12].

**Figure 1:**
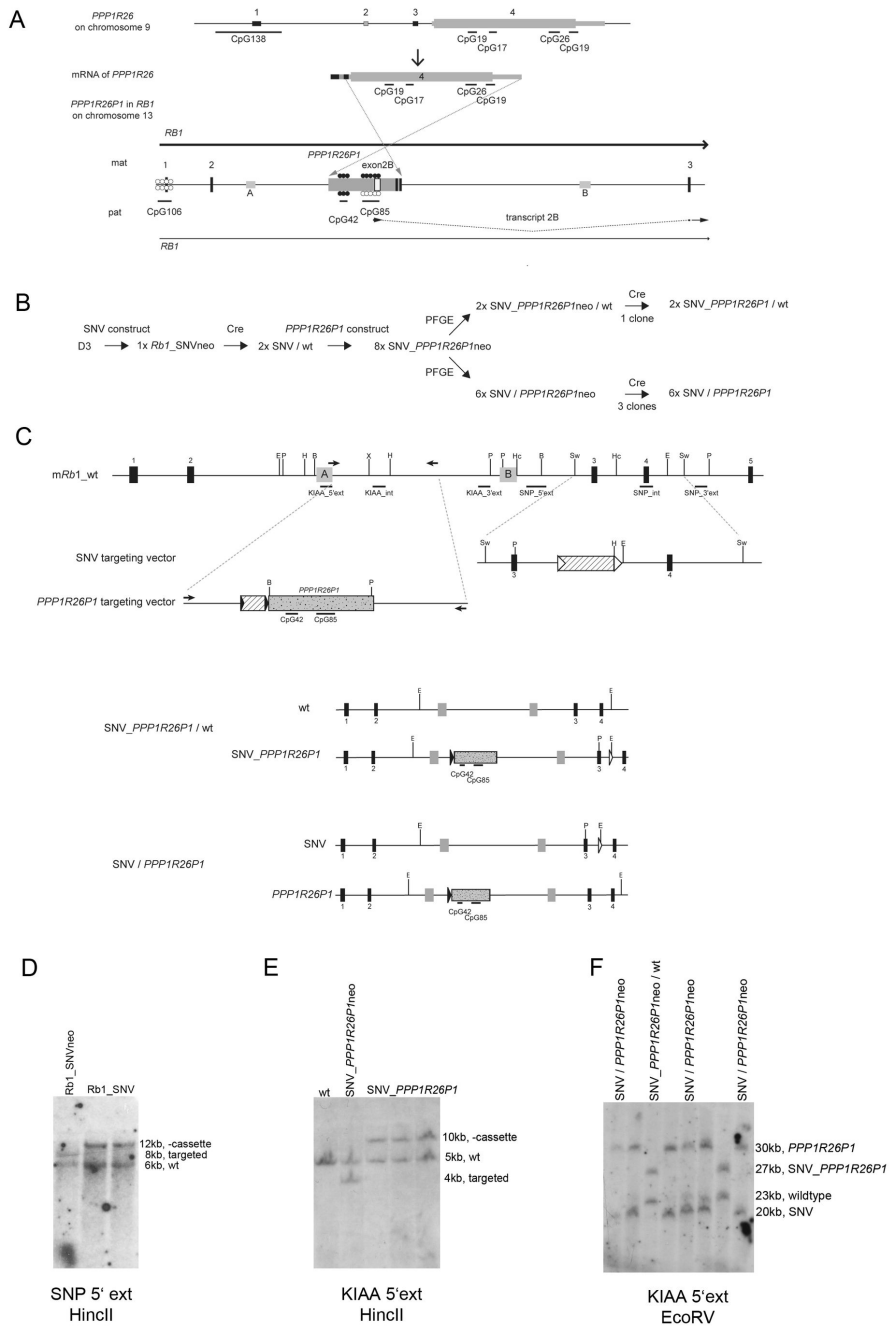
Targeting human *PPP1R26P1* into mouse *Rb1* intron 2. A) Top: structure of the human *PPP1R26* gene on chromosome 9, consisting of four exons; non-coding sequences are indicated by lower box height. Four small CpG islands are represented by black lines below exon 4. Middle: mRNA structure of *PPP1R26*. Below: scheme of exons 1 to 3 (black vertical boxes) of the human *RB1* locus on chromosome 13. Integration of *PPP1R26P1* in intron 2 of *RB1* occurred in inverted orientation and skipping part of exon 1 of the *PPP1R26* mRNA. The pseudogene has two larger CpG islands, CpG42 and CpG85, their methylation status is indicated by circles: filled – methylated, open – not methylated. Expression of *RB1* is skewed in favor of the maternal allele indicated by a thicker arrow. The new exon 2B is shown as white box in *PPP1R26P1*, it splices onto exon 3 of *RB1*. The light gray boxes in intron 2 indicate ECRs A and B. B) Generation of modified ES cells. The order of targeting experiments is shown, indicating the number and designations of clones analyzed. Above the arrows the type of experiment is given: SNV or *PPP1R26P1* construct: introduction of targeting vectors; PFGE: analysis of phasing of the two targeting events; Cre: removal of selection cassette by Cre expression. C) The targeting strategy in murine ES cells. Top: wild type mouse *Rb1* locus on chromosome 14 is shown from exon 1 to 5 (black boxes), also showing the positions of ECRs A and B (grey boxes). Below: constructs used to introduce the SNV (indicated by P for the newly generated PstI restriction site) into exon 3 of *Rb1* (SNV targeting vector) and to introduce human *PPP1R26P1* (gray dotted box, positions of CpG42 and CpG85 shown as black lines) into intron 2 of *Rb1* (*PPP1R26P1* targeting vector). The homology region of the latter was amplified by PCR, primers are indicated as black arrows. Both contracts contained a neomycin selection cassette (striped box) flanked by either loxP (white triangles) or loxP511 (black triangles) sites, respectively. Restriction enzymes: B: BamHI, E: EcoRI, H: HindIII, Hc: HincII, P: PstI, Sw: SwaI, X: XcmI. Southern blot probes are indicated in the top scheme as black lines and labeled. The two possible genomic combinations of targeted alleles are depicted in the lower panel: genotype SNV_*PPP1R26P1*/wt, having both targeting events and the same allele or genotype SNV / *PPP1R26P1* with the two targeting events on different alleles. D) Southern blot analysis of targeted clones carrying the SNV in exon 3 of *Rb1*. Probe SNP 5’ ext hybridized to HincII-digested genomic DNA shows an 8 kb fragment for the targeted allele in *Rb1*_SNVneo and a 12 kb band for the targeted allele after selection cassette removal in *Rb1*_SNV clones. E) Southern blot analysis for targeting of *PPP1R26P1* into intron 2 of *Rb1*. Probe KIAA 5’ ext hybridized to HincII-digested genomic DNA identifies a 4 kb fragment for the targeted allele in the SNV_*PPP1R26P1*neo clone and a 10kb fragment in SNV_*PPP1R26P1* clones after removal of the selection cassette. F) Southern blot analysis of PFGE to determine phasing of the two targeting events. Probe KIAA 5’ ext was hybridised to EcoRV-digested DNA to identify either a 23 kb and a 27 kb band in clones having genotype SNV_*PPP1R26P1*neo/wt (both events on one allele) or a 20kb and a 30kb fragment in clones with genotype SNV / *PPP1R26P1*neo with the two events occurred on different alleles.

In the human *RB1* gene, a processed 5’-truncated transcript of *PPP1R26* was retrotransposed in reverse orientation relative to *RB1* (Figure 1A) [2]. So, in the pseudogene *PPP1R26P1*, the open reading frame of *PPP1R26*, being contained entirely in its exon 4, is in antisense orientation to the *RB1* promoter. Over time, the *PPP1R26* open reading frame in *PPP1R26P1* degenerated and the four small CpG-rich regions in exon 4 of human *PPP1R26* correspond to two larger CpG-islands, CpG42 and CpG85 in the pseudogene copy. CpG42 is fully methylated on both parental alleles, whereas CpG85 presumably acquires DNA methylation in the maternal germline only (Figure 1A). Moreover, CpG85 gained promoter activity and drives expression of an alternative *RB1* transcript, transcript 2B, from the unmethylated paternal allele [2]. This transcript is transcribed in antisense direction relative to the original orientation of transcription in PPP1R26, but in sense direction to *RB1* and splices onto exon 3 of *RB1* (Figure 1A) [2]. Therefore, PPP1R26P1 is not a retrogene, having no function on its own anymore. The development of the new promoter activity in CpG85 is associated with the observed reduction in expression of the full length *RB1* transcript from the paternal compared to the maternal allele. As suggested by Kanber et al., skewed allelic expression may be caused by transcriptional interference [2,13]: recruitment of RNA polymerase II complexes to the CpG85 promoter could block the elongation process of *RB1* transcripts originating at the upstream promoter of full-length *RB1*. Thus, expression of the alternative *RB1* transcript 2B leads to suppression of the full-length *RB1* transcript *in cis* (i.e. on the paternal allele), resulting in about two- to three-fold higher expression from the maternal than from the paternal allele. To our knowledge, *RB1* is the only example of a gene that became imprinted in such a way.

To directly test the influence of *PPP1R26P1* insertion onto the expression of *RB1*, we generated an *in vitro* model of *RB1* imprinted expression by integrating human *PPP1R26P1* into intron 2 of mouse *Rb1* in murine embryonic stem cells (mESCs).

## Results

### Insertion of human *PPP1R26P1* into mouse *Rb1*

Genetic engineering by homologous recombination in mESCs was used to introduce the human *PPP1R26P1* into intron 2 of the non-imprinted mouse *Rb1* gene, positioned and oriented analogous to human *RB1*. To enable detection of allele specific expression, a single nucleotide variant (SNV) was introduced into one of the *Rb1* alleles first. The stepwise generation of the modified ES cells is depicted in Figure 1B. The SNV was inserted into exon 3 using the targeting vector shown in Figure 1C, creating a new PstI restriction site but not altering the encoded amino acid. Successful homologous recombination in wildtype D3 mESCs was screended for by Southern blot. Three clones showed the expected restriction pattern consisting of a 6 kb and an 8 kb fragment for the wildtype and the targeted allele, respectively (Figure 1D). Of these three clones, only one had the SNV integrated in exon 3, as was determined by PCR followed by PstI digestion (Figure S1). A Southern blot hybridized with a probe internal to the targeting vector confirmed that this clone (designated *Rb1*_SNVneo) had a single integration at the *Rb1* locus only (Figure S2A). In addition, a Southern blot on DNA digested with PstI and hybridized with a 3’ external probe, showed that the PstI site was correctly integrated in exon 3 at the *Rb1* locus (Figure S2A). In this *Rb1*_SNVneo clone, removal of the selection cassette generated clones SNV / wt (Figure 1D). Note that the SNV allele is written to the left side throughout the text, this is not related to parental origin of the alleles, as this could not be determined.

One SNV / wt clone was used for the integration of *PPP1R26P1* in intron 2 of *Rb1* at the position corresponding to its location in human *RB1*. The sequences of intron 2 of *RB1* and *Rb1* show no high level of sequence similarity and also have different lengths of 35 kb and 23 kb, respectively. Two evolutionary conserved regions, termed ECR A and B, were identified using the ECR browser (http://ecrbrowser.dcode.org/) (Table S1, Figure 1A, C). In *RB1*, *PPP1R26P1* integrated between these two ECRs, so they were used as anchor points to determine the insertion site in mouse *Rb1*, integrating *PPP1R26P1* at the proportionally correct position between the two ECRs in the same orientation as in human *RB1*.

The targeting construct for *PPP1R26P1*, containing the 5.2 kb *PPP1R26P1* is shown in Figure 1C. Correctly targeted independent clones (designated SNV_*PPP1R26P1*neo) were identified by Southern blot screening (Figure 1E). Phasing of the two recombination events, generation of the SNV in exon 3 and integration of *PPP1R26P1*, was determined in eight SNV_*PPP1R26P1*neo clones by pulsed-field gel electrophoresis followed by Southern blot (Figure 1F). For location of both modifications on the same allele (genotype SNV_*PPP1R26P1*/wt), a 23 kb fragment for the unmodified, wild type allele and a 27 kb fragment for the double-modified allele are expected. In case of the two modifications being located *in trans* (genotype SNV / *PPP1R26P1*), the predicted pattern is a 20 kb fragment for the allele carrying the SNV in exon 3 and a 30 kb fragment for the allele carrying *PPP1R26P1* in intron 2 (Figure 1F). Six clones with genotype SNV / *PPP1R26P1*neo and two clones with genotype SNV_*PPP1R26P1*neo/wt (Figure 1F) were obtained. Three SNV / *PPP1R26P1*neo clones and one SNV_*PPP1R26P1*neo/wt clone were chosen for removal of the selection cassette. Two subclones each were used for further analysis, resulting in six independent clones with genotype SNV / *PPP1R26P1* and two clones with genotype SNV_*PPP1R26P1*/wt in total. All clones were tested by Southern blot for correct, single integration events and successful removal of the selection cassette using 5’ and 3’ external probes, an internal probe and a probe hybridizing to the neomycin gene in the selection cassette (Figure S2B).

### CpG42 and CpG85 have different susceptibilities to DNA methylation

**Figure 2:**
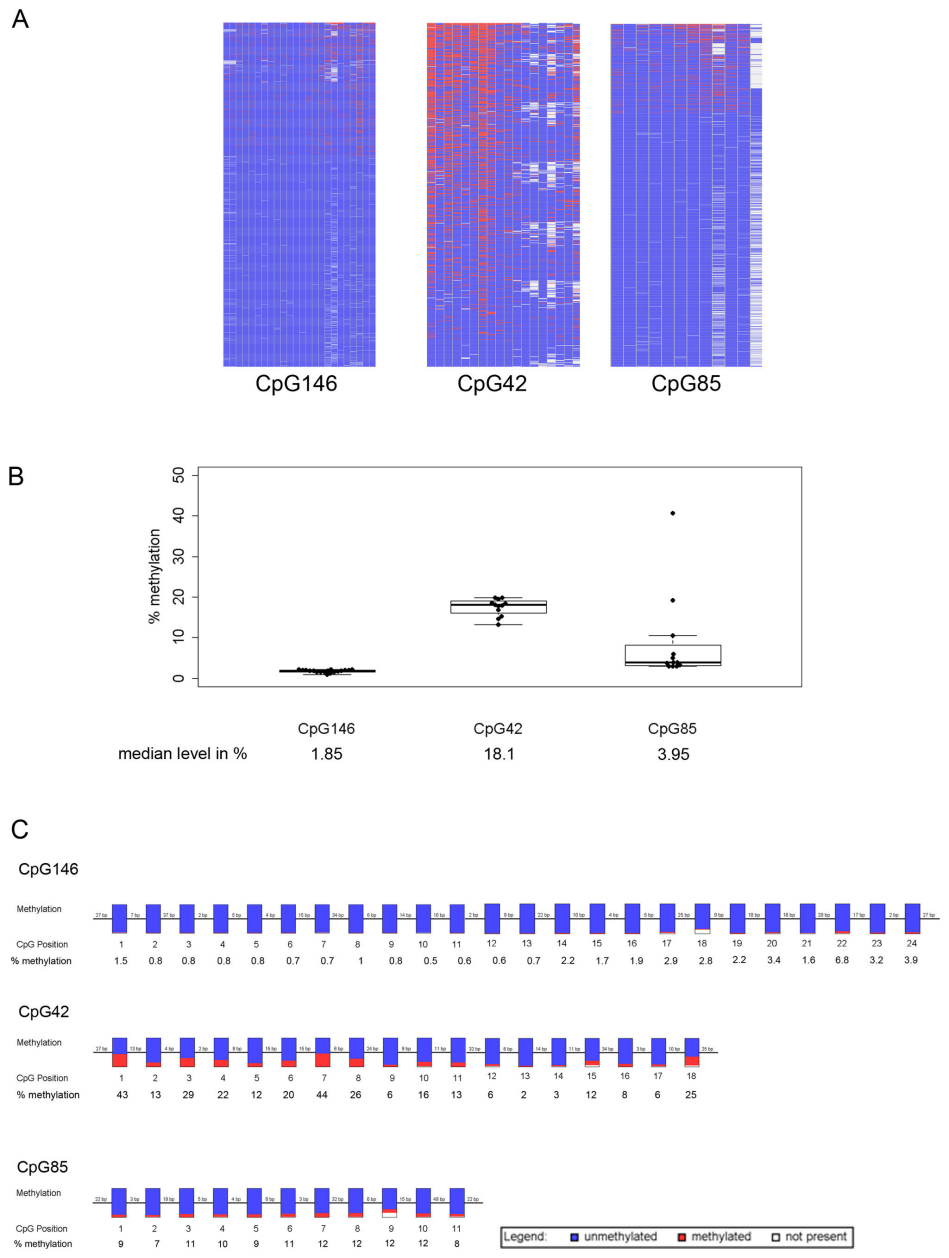
CpG42 and CpG85 have different susceptibility to DNA methylation. A) Differences in DNA methylation levels of the *Rb1* promoter (CpG146) and CpG42 and CpG85 in the integrated *PPP1R26P1* pseudogene on the single read level. One representative DNA methylation heat map for each CpG island is shown. Single reads are in rows, CpG positions in the amplicon are in columns. Red: methylated, blue: unmethylated, white: not analyzed. CpG146 and CpG85 acquire a low level of random DNA methylation, CpG42 acquires DNA methylation at a considerable level, showing preference for certain CpG positions. B) CpG42 has a higher median level of DNA methylation than CpG146 and CpG85. Beeswarm-boxplots show the median percentage of DNA methylation at CpG146, CpG42 and CpG85. The median is calculated over all CpG sites analyzed in all reads for a given CpG island. The boxplots show the median and the quartiles, single measurements are given as black dots. Percentage of median methylation is given below the labels. C) CpG42 has a preference of sites for acquisition of DNA methylation. In pearl-necklace diagrams the degree of DNA methylation per CpG position in all analyzed reads is visualized in histograms. Coloring indicates the percentage of methylated (red), non-methylated (blue) and not-analyzed (white) CpGs. The position of CpG and % of methylation is indicated below the histogram. CpG146 and CpG85 have no preferential CpG position becoming methylated, CpG42 shows strong preference for DNA methylation at CpG positions 1, 7 and a medium preference for CpG positions 3, 8 and 18.

The fate of the two CpG islands introduced with *PPP1R26P1* in the mESCs, CpG42 and CpG85, was analyzed by next-generation bisulfite sequencing (Figure 2, Figure S3A, Figure S4). As control for an unmethylated CpG island, CpG146 at the *Rb1* promoter was included (UCSC genome browser, NCBI37/mm9). DNA methylation on the single read level is visualized in DNA methylation heat maps, showing one sequencing read per row and one CpG position in the amplicon per column. The sequencing reads are sorted from high to low methylation (Figure 2A). The DNA methylation heat maps indicate that CpG146 and CpG85 are sparsely methylated with a random pattern of methylated CpG positions. In contrast, CpG42 acquires DNA methylation to a higher degree and with an apparent preference for certain CpG positions. A summary of the amount of DNA methylation at all CpG positions in all analyzed reads of D3, SNV / wt and SNV / *PPP1R26P1* clones in two biological replicates each shows that the *Rb1* promoter (CpG146) is mostly free of methylation with a median methylation level of 1.85% (Figure 2A, B). The level of DNA methylation at CpG85 was low with a median level of 3.95% (Figure 2A, B). However, two clones showed up with elevated DNA methylation levels of 19% and 40%, respectively. Analysis at the single read level revealed that these two clones mainly contained either fully methylated or fully unmethylated reads (Figure S4A), indicating the presence of a mixed population of cells with some cells having a fully methylated CpG85 in their single *PPP1R26P1* allele. At CpG42, the degree of total methylation is about five-fold higher than at CpG85 with a median level of 18.1% (Figure 2A, B).

The degree of methylation at a specific CpG position in the amplicon over all reads analyzed is visualized in the pearl-necklace diagram (Figure 2C). This shows that CpG146 and CpG85 have no preferential CpG position for acquisition of DNA methylation. For CpG42, the pearl-necklace diagram shows preferential DNA methylation at positions 1 and 7 of the CpG sites included in the amplicon, which are methylated in 40% of analyzed reads. The CpG sites at positions 3, 8 and 18 in the amplicon are methylated in more than 25% of reads. As methylation at these positions could serve as seed for acquisition of full DNA methylation at this CpG island, methylation levels were determined again in four clones at 15 passages later, representing about 30 to 40 cell divisions. However, the methylation level at CpG42, and at CpG146 and CpG85 as well, was stable over this time period and did not spread over the full CpG island (Figure S4B).

Analysis of two clones with the genomic arrangement SNV_*PPP1R26P1*/wt showed no difference in median methylation levels compared to the six clones with the genotype SNV / *PPP1R26P1*, indicating that the phase of the introduced modifications does not influence the DNA methylation levels at the three CpG islands analyzed (Figure S3A).

### Transcription at CpG85 in mouse *Rb1*

**Figure 3:**
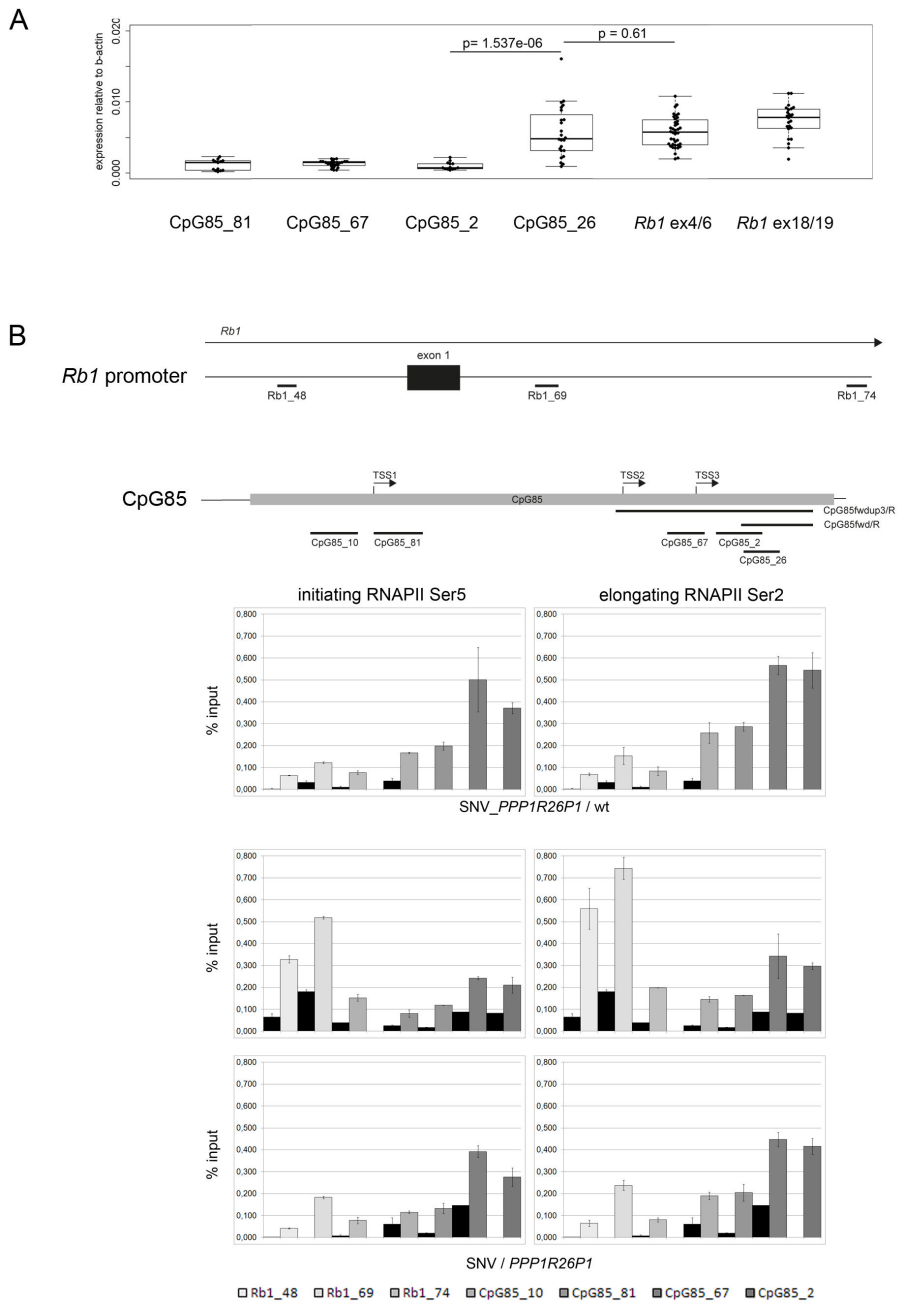
Transcription at CpG85. A) Expression of the transcript is elevated towards the 3’-end of CpG85. Quantitative RT-PCR with primer / probe assays (localization is shown in Figure 3B top) demonstrates significantly higher expression at the more 3’-end of CpG85. For comparison, expression of *Rb1* is shown as measured with assays at exons 4/6 and exons 18/19. Analysis was conducted in six independent clones in two biological and two technical replicates, respectively. Expression levels are normalized to the level of β-actin. The p-value was calculated using the Welch two-sample test. B) RNA polymerase II (RNAPII) is enriched at the 3’ end of CpG85. Presence of initiating and elongating RNAPII at the *Rb1* and CpG85 promoters was tested for by ChIP. Top: schemes showing localization of primer / probe assays for qPCR over the *Rb1* promoter (three assays) and CpG85 (four assays), as well as the mapped transcriptional start sites in CpG85. Results are depicted as % input and the non-related antibody (against PML protein) control is shown in black. Both types of RNAPII could be found at both promoters. Three independent experiments are shown, one in cells with genotype SNV_*PPP1R26P1*/wt and two in independent clones of genotype SNV / *PPP1R26P1*.

In humans, expression of the alternative *RB1* transcript 2B starts from an unmethylated CpG85 within *PPP1R26P1*. As we could show that CpG85 is largely unmethylated in the modified ESCs, expression of a transcript from CpG85 was measured. A transcript starting at the 3’-end of CpG85 could be amplified by standard RT-PCR. Using primers in CpG85, as depicted in Figure 3B, a transcript was detected with forward primers CpG85fwd_up3 and CpG85_fwd, but not with primers located further upstream (Figure S5). Expression of *Rb1* and the house keeping gene *Ppia* was present in all samples (Figure S5). Quantitative RT-PCR revealed an elevated, but highly variable level of transcription at the position of probe CpG85_26 compared to probes CpG85_81, CpG85_67 and CpG85_2 (Figure 3A). The level of transcription measured with probe CpG85_26 was lower but comparable to the expression levels observed for the full-length *Rb1* transcript, measured with probes spanning exons 4 to 6 and exons 18 to 19 (Figure 3A). The transcription start site of the detected transcript could not be determined, as 5’-RACE (rapid amplification of cDNA ends) experiments failed. In addition, no transcript connecting transcript 2B to downstream exons of *Rb1* was detected by RT-PCR. Use of reverse primers in exons 3, 5 and 8 did not yield a product. Also, attempts to elongate the obtained PCR products by 3’-RACE failed.

Binding of initiating and elongating RNA polymerase II (RNAPII) at the 3’-end of CpG85 could be shown by chromatin immunoprecipitation (ChIP) in clones with the genotype SNV / *PPP1R26P1* as well as the genotype SNV_*PPP1R26P1*/wt (Figure 3B). Enrichment of initiating RNAPII was more pronounced at the 3’- end of CpG85, corresponding to the data obtained by qPCR. Precipitation with an antibody against elongating RNAPII showed a similar pattern with enhanced enrichment at the 3’-end of CpG85. RNAPII occupancy of the *Rb1* promoter in the same clones was determined as control. The obtained pattern was again similar for antibodies against the initiating and elongating forms of RNAPII. Specificity was confirmed with an antibody against promyelocytic leukemia protein (PML), showing no enrichment of both RNAPII types over the *Rb1* promoter or CpG85 (Figure 3B). These results indicate active transcription at the 3’-end of CpG85. Cells with the genotype SNV_*PPP1R26P1*/wt showed the same result, with enhanced expression of transcript 2B and enrichment of initiating and elongating RNAPII at the 3’-end of CpG85 (Figure 3B, S3B).

### Integration of *PPP1R26P1* suppresses *Rb1 in cis*

**Figure 4:**
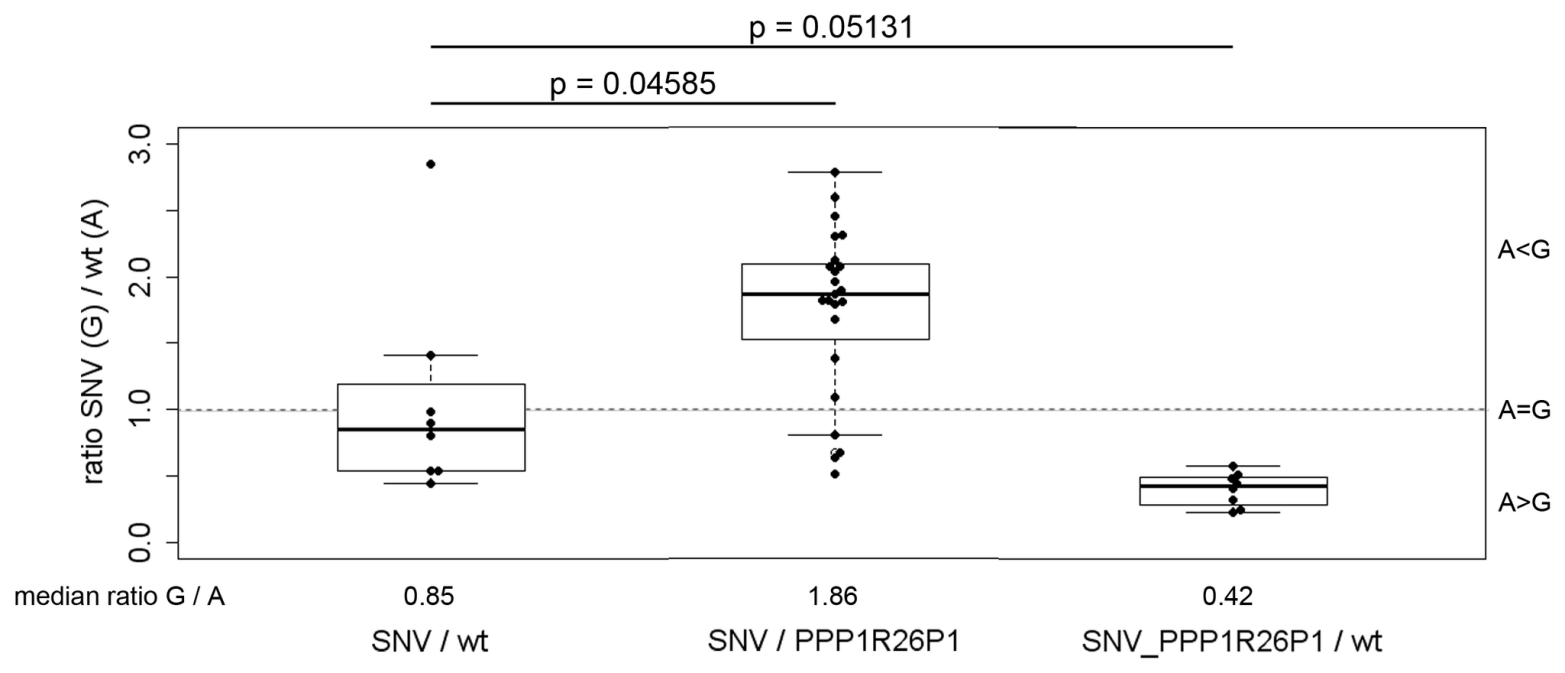
Integration of *PPP1R26P1* represses *Rb1 in cis*. Single nucleotide primer extension analysis (SNaPshot) was used to determine the ratio of wildtype versus SNV allele expression in six clones with genotype SNV / *PPP1R26P1* and two clones with genotype SNV_*PPP1R26P1*/wt in two biological and two technical replicates. Obtained value*s* were normalized to values obtained for genomic DNA of the same clones. Beeswarm boxplots show the median and the quartiles, single measurements are given as black dots. The dotted line indicates the level expected for non-skewed expression. In SNV / wt cells, both alleles express about equal levels of *Rb1*. In cells with the genotype SNV / *PPP1R26P1*, this ratio shifts to preferential expression of the SNV allele (G) and suppression of the wildtype A allele carrying the *PPP1R26P1* insertion. Cells with genotype SNV_*PPP1R26P1*/wt again show suppression of *Rb1* on the allele carrying *PPP1R26P1* (the G allele) versus the wildtype A allele. P-values were calculated using the Welch two-sample test.

As CpG85 remains mainly unmethylated and a transcript is initiated at the 3’-end of CpG85, just as in human *RB1*, skewing of allelic *Rb1* expression was tested by single nucleotide primer extension (SNaPshot) analysis. The SNaPshot assay was validated using plasmids containing either an A or a G at the variant position (data not shown). Analysis of SNV / wt cells showed roughly balanced expression of the two alleles (Figure 4, left). In SNV / *PPP1R26P1* cells carrying the *PPP1R26P1* on the wildtype A allele, expression of this allele is suppressed, resulting in an about two-fold shift of expression in favor of the SNV G allele (Figure 4, middle). Suppression of expression of the allele carrying the *PPP1R26P1* insertion was also observed in the SNV_*PPP1R26P1*/wt cells. In these cells, which carry the *PPP1R26P1* insertion on the variant G allele, a two-fold stronger suppression of this allele was observed (Figure 4, right).

## Discussion

The use of mESCs becomes more and more accepted for studying the mechanisms of imprinted gene expression and its regulation [14–16]. The capability of mESCs to be genetically modified by homologous recombination and to be differentiated into several lineages makes them a useful tool to dissect imprinting mechanisms during development and to modify and study the genetic elements involved, as was exemplified at the murine *Igf2r/Airn* locus [15,17–19]. Most importantly, by the use of mESCs it could be demonstrated recently that it is the transcriptional overlap of *Airn* with the *Igf2r* promoter and not the *Airn* transcript itself that induces *Igf2r* silencing [20], establishing transcriptional interference as imprinting mechanism [21]. Our study at the *Rb1* locus expands the use of mESC models to test the relevance of genetic elements, like pseudogenes, for imprinted gene expression across species.

The integration of human *PPP1R26P1* into mouse *Rb1* in mESCs correlates with reduced levels of *Rb1* transcripts *in cis*, identifying the unmethylated *PPP1R26P1* as a *cis*-repressive element. Expression of the allele carrying *PPP1R26P1* was about half as compared to the wild type allele (Figure 4). This is comparable to the level of skewing observed in human samples, varying from two- to about three-fold [2]. The decrease in *Rb1* expression was accompanied by expression of a transcript originating at the unmethylated CpG85 in *PPP1R26P1*. This transcript is relatively short and it is enriched at the 3’-end of CpG85. The precise transcriptional start of the transcript could not be determined so far. But the use of forward primers locating further upstream in the CpG island did not yield a product, indicating that the observed transcript is not due to the amplification of an intron-containing full-length *Rb1* transcript. This assumption is supported by the qPCR data (Figure 3A). Further we could show by ChIP that initiating RNA polymerase II is binding to CpG85, supporting transcription initiation at CpG85 (Figure 3B). Elongating RNA polymerase II was detected at the *Rb1* promoter and CpG85 as well. However, here we cannot distinguish if the signal relates to full length *Rb1* transcription or to transcripts starting at CpG85. Unlike in human *RB1*, we could not detect splicing of the transcript onto downstream exons of *Rb1*. This could indicate that transcription at CpG85 in mouse *Rb1* might not switch into efficient elongation, thus resulting in short transcripts not connected to downstream exons of *Rb1*.

Despite the difference in length of human transcript 2B and the detected murine transcript, its expression was associated with repression of the full-length *Rb1* transcript *in cis*. This suggests that the act of transcription or merely transcription initiation at the CpG85 promoter might be relevant for the repression of *Rb1* transcripts, rather than the length of the transcript. Transcriptional interference is defined as one transcription process repressing another transcription process *in cis* [13]. At the human *RB1* locus an active downstream CpG85 promoter interferes with transcript production from the upstream *RB1* promoter, which could be explained by the roadblock model of transcriptional interference, as proposed by Kanber et al. [2]. The same would apply to the *Rb1*_*PPP1R26P1* allele present in our mESC model. According to the roadblock model, the progression of RNAPII transcribing full-length *RB1* is inhibited by binding of another RNAPII or transcription factors at the tandem downstream CpG85 promoter located within the transcription unit of *RB1* [13]. A consequence of transcriptional interference at CpG85 could be premature termination of *RB1* / *Rb1* transcripts upstream to *PPP1R26P1*, leading to an underrepresentation of full-length *RB1* / *Rb1* transcripts from the paternal allele. The use of alternative polyadenylation (poly(A)) sites depending on the activity of promoters in intronic retroelements was described at the *Cabp* ^*IAP*^ allele of the *Cabp* gene and the imprinted ‘intronic-host’ pair *Mcts2/H13* [8,11,22]. Active transcription in sense orientation from the unmethylated retroelement resulted in premature termination of the host gene at alternative or cryptic poly(A) sites upstream of the retroelement. However, premature termination of *RB1* upstream of *PPP1R26P1* seems unlikely, as a search for poly(A) sites using the UCSC browser (assembly NCBI36/hg18) did not reveal any reported or predicted poly(A) sites upstream of *PPP1R26P1* in the *RB1* gene. It is noteworthy that transcription interference could also provide an explanation for the low level of transcription of transcript 2B, and maybe, the premature termination of transcription observed in the murine model system: transcription from the upstream *Rb1* promoter could interfere with transcription at the downstream CpG85 promoter.

Transcription initiation at CpG85 relies on an unmethylated CpG island in humans and mESCs. By next generation bisulfite sequencing we showed a low level of random DNA methylation at CpG85. In contrast, CpG42 acquired methylation at considerable level with preference for certain CpG positions (Figure 2). As the mESC clones under study did not pass through the mouse germline and contain *PPP1R26P1* on one allele only, we did not expect to observe differential DNA methylation on CpG85. However, the differences we see in DNA methylation acquisition at CpG85 in comparison to CpG42 located in the same retroelement might reflect inherent features of these two CpG islands. In humans, CpG42 and CpG85 belong to different classes of CpG islands, as CpG85 is a differentially methylated region and CpG42 is not: in blood, CpG85 is methylated on the maternal allele only, whereas CpG42 is fully methylated on both parental alleles [2]. Tucker et al. showed that gametic differentially methylated regions, like CpG85, are dependent on germline transmission to acquire DNA methylation, as they are resistant to DNA methylation in ES cells [23]. Therefore it is not surprising that CpG85 remains unmethylated in our mESC model. Recent whole-genome analyses of mouse germ cells underlined the observation that a far higher number of CpG islands becomes methylated in oocytes than in sperm, and that transcription through CpG islands plays a role in establishing DNA methylation [20,24–27]. Proudhon et al. highlighted that a discrimination should be made between imprinted and transient gametic differentially methylated regions: both types are differentially methylated in the parental germ lines, being methylated mainly in the oocyte, and are maintained during the global wave of demethylation during preimplantation development, but only a true imprinted gametic differentially methylated region will be protected against *de novo* acquisition of DNA methylation on the unmethylated allele [5]. The persistence of gametic differentially methylated regions against *de novo* methylation might be tissue-specific, showing leveling of differential methylation in some tissues but not in others [5,28,29]. In humans, the methylation status of CpG85 and CpG42 could not be determined in oocytes but both CpG islands are unmethylated in sperm ( [2] and D. Kanber, unpublished). Therefore we cannot exclude that both CpG islands become methylated in the human oocyte, but only CpG85 is protected against *de novo* DNA methylation during development, leading to the observation of differential methylation in blood [2]. The different levels of methylation observed for CpG42 and CpG85 in the mESC model might reflect this difference in the resistance to *de novo* methylation in postimplantation development. Of note, we observed gain of DNA methylation on CpG85 in several tissues, obliterating the degree of differential methylation on this differentially methylated region (Kanber et al., manuscript in preparation). This observation in human tissues might be reflected by the finding of elevated DNA methylation levels at CpG85 in two clones, indicating that, in principle, CpG85 can be methylated to a high extent. In contrast to the stability of low level DNA methylation at CpG85, the high level of DNA methylation observed in some clones was lost during *in vitro* culture. Probably those cultures showing high levels of CpG85 methylation contained a relatively high amount of differentiated cells, which was selected against during subsequent culture.

In conclusion, in our *in vitro* mESC model of *RB1* imprinted expression, we show repression of *Rb1* expression *in cis* by insertion of the human pseudogene *PPP1R26P1* into mouse *Rb1*. This is associated with an unmethylated state of CpG85 and observation of a short transcript at CpG85. The modified ES cells will be used for the generation of mice, transferring the model to the *in vivo* situation. This allows to study if *PPP1R26P1* acquires gametic DNA methylation upon transmission through the germline. The animals will be used also to study the influence of pseudogene integration on the development of tumors, with a special emphasis on parent-of-origin effects, which are observed in human retinoblastoma [30,31].

## Materials and Methods

### ES cell culture and gene targeting

Murine D3 ES cells (provided by Denise P. Barlow, CeMM, Vienna) were maintained on inactivated feeder cells under standard cell culture conditions, using KO-DMEM supplemented with 1/100 penicillin/streptomycin, 1/100 glutamine, 2 µM β-mercaptoethanol, 15% FCS, 1/100 non-essential amino acids, 1/1000 LIF (all ingredients from Life Technologies). Cells were kept in a humidified incubator at 37°C with 5% CO_2_ and subcultured every second to third day.

For gene targeting, 5x10^6^ cells were electroporated with 50 µg linearized targeting vector using the BioRad electroporation device at 500 µF and 0.24 kV. After electroporation, cells were plated onto resistant feeder cells at a density of 1x10^6^ cells per 10 cm plate and selection with 400 µg/ml G418 (Roche Applied Science) started 24 h after electroporation, lasting for 7 days. Single resistant clones were picked, subcultured, frozen and screened by Southern blot. Positive clones were thawed and expanded, retested by Southern blot and the neomycin selection cassette was removed by Cre recombination using electroporation of 50 µg circular Cre expression plasmid. Cells were plated at low density; single clones were picked, expanded and tested by Southern blot for selection cassette removal. Introduction of the SNV was performed first (509 clones screened with probe SNP5’ext, targeting efficiency was 0.6%), followed by *knock-in* of *PPP1R26P1* (452 clones screened with probe KIAA5’ext, targeting efficiency was 39%).

### Construction of targeting vectors

The targeting vector for introduction of the SNV consists of a 6.5 kb homology region generated by SwaI digestion of BAC bMQ311j15 (obtained from Source BioScience, UK) (UCSC browser, build NCBI37/mm9, chr14:73,692,400-73,698,980), ligated into pBluescript opened with HincII. Before choosing exon 3 for integration of the SNV, absence of sequence motifs indicating exonic splice enhancers was confirmed using ESEfinder 3.0 (http://rulai.cshl.edu/cgi-bin/tools/ESE3/esefinder.cgi [32]) to minimize the risk of aberrant splicing as a result of the modification. For introduction of the SNV, a fragment of 1.2 kb was generated by PCR using BAC bMQ311j15 as template and forward and reverse primers containing a PmeI and a BsiWI restriction site, respectively. The obtained PCR product was ligated into TOPO-vector pCR2.1 (Life Technologies). This vector was used as template in the mutagenesis reaction in combination with appropriately designed primers using the QuikChange II XL site-directed mutagenesis kit (Agilent). Nucleotide 96 in exon 3 of *Rb1* (total size 116 nucleotides) was changed from A to G, creating a new PstI site. Successfully mutated plasmids were identified by PstI digestion and verified by sequencing. The 1.2 kb fragment was reintroduced into the homology vector using restriction enzymes PmeI / BsiWI, resulting in pBS_SNV. After pBS_SNV was linearized with HindII, the neomycin selection cassette, consisting of a loxP_PGK/neo_loxP array, was introduced into intron 3, resulting in the SNV targeting vector. This vector was linearized by NotI digestion before electroporation.

To determine the insertion site of *PPP1R26P1* into mouse *Rb1*, the distances between ECR A and the 5’ end of *PPP1R26P1* and between the 3’ end of *PPP1R26P1* and ECR B were calculated as proportion of the total intronic sequence between the two ECRs. The nucleotides representing this percentage in the mouse intron between the two ECRs were calculated. The targeting vector for the introduction of *PPP1R26P1* contains a 6.6 kb homology fragment (UCSC browser, build NCBI37/mm9, chr14:73,707,316-73,713,900) generated by long-range PCR using the Phusion Flash high-fidelity PCR master mix (Thermo Scientific) and BAC bMQ311j15 as template. XhoI and NotI restriction sites were introduced with the long-range PCR primers. The fragment was ligated into the vector pACYC_D4 opened by XhoI and NotI and sequence-verified by Sanger sequencing, generating pACYC_D4_ECR. *PPP1R26P1* was generated by long-range PCR using the Phusion Flash high-fidelity PCR master mix (Thermo Scientific) using BAC RPCIB753O20259Q (obtained from imaGenes) as template and introducing SwaI restriction sites for cloning into pACYC_D4_ECR and the introduction of the neomycin selection cassette. Integrity of *PPP1R26P1* was verified by Sanger sequencing. *PPP1R26P1* was introduced into the unique XcmI site of pACYC_D4_ECR. The selection cassette, consisting of a loxP511_PGKneo_loxP511 array, was introduced directly upstream of *PPP1R26P1* using the EcoRV site introduced by the PCR forward primer. The targeting vector was linearized by NotI before electroporation.

### Isolation of genomic DNA and RNA

Prior to isolation of genomic DNA and RNA of successfully targeted ES cell clones, ES cells were cultivated feeder-free on gelatinized dishes for three passages.

Genomic DNA was isolated by alkaline lysis using standard methods [33]. RNA was isolated using QIAzol (Qiagen) and the standard protocol provided by the manufacturer.

### Southern blot and PFGE

Restriction enzyme digests for Southern blot were performed with HincII, HindIII or BamHI enzymes for detection by SNP and KIAA 5’ external probes. PstI was used for SNP and KIAA 3’ external and internal probes, and BamHI for the neo probe (all restriction enzymes from New England Biolabs). Southern blotting was performed according to standard procedures by capillary transfer [33]. Probes were radioactively labeled using the Megaprime kit (GE Healthcare) and blots were hybridized overnight and washed at 65°C with Church buffer. PCR primers for probe generation are listed in Table S2.

To analyze phasing of the two targeting events, pulsed-field gel electrophoresis (PFGE) using the CHEF Mapper XA system (BioRad) was applied. Genomic DNA was digested with EcoRV and resolved on a 0.8% TBE gel in 0.5x TBE. Settings were lower limit 15 kb, upper limit 35 kb and run time 27 h 18 min. After electrophoresis, Southern blotting and hybridization was done as described using the KIAA 5’ external probe.

### Next generation bisulfite sequencing

Next generation bisulfite sequencing on the Roche 454 Junior system was carried out as described [34]. Briefly, bisulfite conversion of genomic DNA was performed using the EZ DNA Methylation-Gold Kit (Zymo Research) according to the manufacturer’s instructions. Converted DNA was amplified in a first round of PCR using tagged primers and the Qiagen HotStarTaq Master Mix (Qiagen). In a second round of PCR, sample-specific bar code sequences (MIDs – multiplex identifiers) and universal linker adapters for the 454 system were added using primers complementary to the first-round PCR tags. Amplicon libraries were purified, diluted, clonally amplified by emulsion PCR and sequenced on the Roche 454 GS junior system according to the manufacturer’s protocol.

Data analysis included stringent quality score filtering, sorting according to MIDs and separation of amplicons with same MIDs using the Geneious 6 software (Biomatters) and methylation analysis using the BiQAnalyzer HT (http://biq-analyzer-ht.bioinf.mpi-inf.mpg.de [35]). Raw data are available on request. The mean methylation over all CpGs analyzed in all amplicons in all clones studied, the number of analyzed reads and the bisulfite conversion rate are listed in Table S3. The bisulfite conversion rate was on average 96.3%, 96.6% and 88% for CpG146, CpG42 and CpG85, respectively.

### RT-PCR and qPCR

RNA was digested with RNase-free DNase I RQ1 (Promega) and 5 µg were reverse transcribed into cDNA using the GeneAmp RNA PCR kit (Applied Biosystems) with random hexamers. PCR was performed using GoTaq polymerase (Promega) under following conditions: 95°C 2 min, 35 cycles: (95°C 30 sec, annealing temperature 58-62°C for 30 sec, 72°C 30 sec), 72°C 5 min, hold at 4°C. Amplification products were visualized by gel electrophoresis. Primer sequences are listed in Table S2.

Quantitative PCR was conducted on the LightCycler 480 II (Roche Applied Science) using the LightCycler480 probes master mix with corresponding primers and universal probe library (UPL) probes (Roche Applied Science). Primer / probe combinations are listed in Table S2. Cycling conditions were 96°C 1 min, 45 cycles: (96°C 10 sec, 60°C 15 sec, 72°C 1 sec), hold at 4°C.

### SNaPshot analysis

SNaPshot analysis for determination of allelic ratios of mRNA (after reverse transcription into cDNA) and genomic DNA as reference was conducted using the ABI Prism SNaPshot ddNTP Primer extension kit (Applied Biosystems) according to the manufacturer’s instructions. The reaction products were analyzed on an ABI 3700 sequencer (Applied Biosystems) and electropherograms were analyzed using Gene Mapper 4.0 software (Applied Biosystems). Allelic ratios of cDNA were normalized to ratios obtained with genomic DNA from the same sample. Primer sequences are listed in Table S2. Two, two and six clones in two biological and two technical replicates each were included in the analysis for SNV / wt, SNV_*PPP1R26P1*/wt and SNV / *PPP1R26P1*, respectively.

### Chromatin immunoprecipitation

Chromatin immunoprecipitation (ChIP) was carried out on formaldehyde-fixed chromatin of modified and wildtype ES cells. Cells were harvested by trypsinization and adjusted to 1x10^7^ cells in 500 µl PBS, and crosslinked using formaldehyde at 1% final concentration for 10 min at room temperature with agitation. Glycine was added up to 0.125 M final concentration to stop crosslinking and cells were incubated at room temperature for 5 minutes with agitation. Cells were washed once with cold PBS and once with ChIP lysis buffer (20 mM Tris-Cl pH8.0, 85 mM KCl, 0.5% NP-40). Cell pellets were snap-frozen in liquid nitrogen and stored at -80°C until further use. Cell pellets were thawed on ice and resuspended in 1 ml ChIP lysis buffer, incubated for 10 min at 4°C with agitation. Cells were pelleted by centrifugation (250g, 5 min, 4°C) and resuspended in nuclei lysis buffer (50 mM Tris-Cl pH8.0, 10 mM EDTA, 1% SDS). Chromatin was sheared using the Covaris S220 instrument with 6x16mm microtubes (Covaris) at following conditions: duty cycle 5%, intensity 4, peak incident power 140 Watts, 200 cycles per burst, time 3 min. After shearing, samples were centrifuged (18,000g, 4°C, 10 min) to remove unsoluble debris. 20 µl of sheared chromatin were used for reverse crosslink and analysis of fragment size by gel electrophoresis. Fragment size ranged from 200 to 600 base pairs. For immunoprecipitation 50 µl of sheared chromatin was set aside as input and 20-40 µg of sheared chromatin in a volume of 100 µl was used for antibody incubation. The chromatin was diluted 1:10 with dilution buffer (20 mM Tris-Cl pH8.0, 150 mM NaCl, 2 mM EDTA, 1% Triton X-100) and precleared with 50 µl of diluted Dynabeads Protein A (Life Technologies) for 1 h at 4°C with agitation. Before use, Dynabeads were washed three times with dilution buffer and blocked with 1 mg/ml BSA overnight. Per sample, 4 µg of antibody were added and incubated overnight at 4°C with agitation. Antibodies used were: rabbit polyclonal to PML (abcam #ab53773) as non-related control, rabbit polyclonal to RNAPII CTD repeat YSPTSPS (phospho S5) (abcam #5131 or Bethyl laboratories # A300-655A), rabbit polyclonal to RNAPII CTD repeat YSPTSPS (phospho S2) (abcam #5095). Antibody-bound chromatin was coupled to blocked Dynabeads for 4 h at 4°C with agitation. Afterwards, beads were washed once with 1 ml of each of the following buffers: low salt buffer (20 mM Tris-Cl pH8.0, 2 mM EDTA, 150 mM NaCl, 0.1% SDS, 1% Triton X-100), high salt buffer (20 mM Tris-Cl pH8.0, 2 mM EDTA, 500 mM NaCl, 0.1% SDS, 1% Triton X-100), LiCl buffer (20 mM Tris-Cl pH8.0, 1 mM EDTA, 250 mM LiCl, 1% NP-40, 1% Sodiumdeoxycholate), and twice with TE buffer (10 mM Tris pH8.0, 1 mM EDTA). Each cycle of washing was conducted for 10 min at 4°C with agitation. After washing, beads were resuspended in 200 µl elution buffer (50 mM Tris-Cl pH8.0, 10 mM EDTA, 1% SDS) and incubated for 45 min at 65°C at 700 rpm in a thermomixer (Eppendorf). The input sample was diluted with elution buffer up to 200 µl final volume. Afterwards, supernatant was transferred to a new tube and 10 µl of proteinase K (10 mg/ml) was added to precipitated samples and input for incubation overnight at 55°C and 300 rpm in a thermomixer. Then 1 µl of RNase A (10 mg/ml) was added and samples were incubated at 37°C for 1 h. Samples were extracted with the MinElute reaction cleanup kit (Qiagen) and eluted in 20 µl buffer. qPCR analysis was performed using 1 µl of eluted chromatin and input (diluted 1/20 before) as template. Primer/probe combinations are listed in Table S2. Results are calculated and represented as % input.

## Supporting Information

Figure S1 **Analysis of *Rb1_SNV*/wt clones for presence of the SNV.** Genomic DNA of three independent, correctly targeted (as judged by Southern blot) Rb1_SNVneo clones was amplified by PCR with the indicated, Rb1 exon 3 flanking, primers and the obtained product was digested with PstI. Presence of the introduced SNV results in a PstI restriction site in the amplified fragment. Digestion yields a 1338 bp fragment for the untargeted wild type allele and 718 bp and 620 bp fragments for the targeted SNV allele. Only clone 197 contains the SNV.
(TIF)

Figure S2 **Southern blots of targeted ES cell clones.** A) Analysis of the SNV allele by Southern blot. Positions of Southern blot probes are indicated in Figure 1C. left panel: genomic DNA of *Rb1*_SNVneo #197 and two SNV / wt clones was digested with PstI and the blot hybridized with the SNP 3’ external probe; expected bands for correctly targeted clones: 12 kb for the wild type allele, 6 kb for the targeted allele in *Rb1*_SNVneo and 6 kb for the targeted allele after removal of the selection cassette in SNV / wt clones. middle panel: genomic DNA of four independently targeted SNV / *PPP1R26P1* clones was digested with BamHI and the blot hybridized with the SNP internal probe; expected bands for correctly targeted clones: 8 kb for the wild type allele, 10 kb for the targeted allele and 8 kb for the targeted allele after removal of the selection cassette. Occurrence of only one band indicates a single targeting event in the analyzed clones.
B) Analysis of *PPP1R26P1*neo allele by Southern blot. Eight independently targeted SNV_*PPP1R26P1*neo clones were analyzed. left panel: genomic DNA digested with PstI and hybridized with the KIAA 3’ external probe is expected to result in : 12 kb for the wild type allele and 8 kb for the targeted allele. middle panel: genomic DNA digested with PstI and hybridized with the KIAA internal probe; expected bands for correctly targeted clones: 12 kb for the wild type allele and 8 kb for the targeted allele. Presence of the indicated two fragments indicates a single targeting event in the genome. right panel: genomic DNA was digested with BamHI and the blot hybridized with the neo probe recognizing the neomycin selection cassette; expected band for correct and single targeted clones: 3 or 5 kb, depending on the orientation of the selection cassette in the targeting vector.
(TIF)

Figure S3 **Analysis of two independent clones with genotype SNV_*PPP1R26P1*/wt.** A) DNA methylation at CpG islands CpG146, CpG42 and CpG85. The median percentage of DNA methylation calculated over all CpG positions in all analyzed reads is indicated. Measurements for clones with genotype SNV_*PPP1R26P1*/wt are given on the left hand side for each CpG island, on the right hand side measurements presented in Figure 2B for genotype SNV / *PPP1R26P1* are depicted for better comparability. No differences in the susceptibility to DNA methylation at all three CpG islands were observed.
**B**) Transcript expression from CpG85. Quantitative RT-PCR using the assays located in CpG85 (as indicated in Figure 3B) show higher expression towards the 3’-end of CpG85 in cells with genotype SNV_*PPP1R26P1*/wt. This is in accordance to the results obtained for cells with genotype SNV / *PPP1R26P1* (shown in Figure 3A).
(TIF)

Figure S4 **Analysis of DNA methylation in later passage cells.** A) DNA methylation heat maps of next generation bisulfite sequencing of CpG85. One clone of genotype SNV_*PPP1R26P1*/wt and two clones of genotype SNV / *PPP1R26P1* were analyzed for DNA methylation at CpG85 after one freezing and thawing (middle panel) cycle and after prolonged passaging (right panel). High transient DNA methylation at CpG85 can be observed at the initial passages (P53, P55; left panel), but it is lost after one freezing and thawing cycle (P57, P58; middle panel) and not stable during continuous culture up to passage 70 (right panel). The median percentage of DNA methylation over all CpG sites in all reads is given below the DNA methylation heat maps. Red: methylated, blue: unmethylated, white: not analyzed.
**B**) Median levels of DNA methylation at CpG146, CpG42 and CpG85 at passage 70 measured in four independent clones with genotype SNV / *PPP1R26P1*. DNA methylation was analyzed by next generation bisulfite sequencing. By comparison to the levels of DNA methylation shown in Figure 2B, the median level (given below the labels) of DNA methylation remains stable over the employed 15 passages, being equivalent to about 30 cell divisions.
(TIF)

Figure S5 **Transcript expression at CpG85.** Standard RT-PCR shows expression of a transcript at the 3’-end of CpG85. Expression was detectable using two RT-PCR assays, detecting transcripts from TSSs 2 and 3 in four independent SNV / *PPP1R26P1* clones but not in SNV / wt cells. Expression of *Rb1* and the house keeping gene *Ppia* was detectable in all cell clones.
(TIF)

Table S1 **Characteristics of evolutionary conserved regions A and B.**
(XLSX)

Table S2 **Primer sequences.**
(XLSX)

Table S3 **Data of next generation bisulfite sequencing.**
(XLSX)
