# Supplementary figures and images for: Human PPP1R26P1 Functions as cis-Repressive Element in Mouse Rb1
Source: PLoS One. 2013 Sep 3;8(9):e74159. doi: 10.1371/journal.pone.0074159 (PMC3760807; doi:10.1371/journal.pone.0074159)

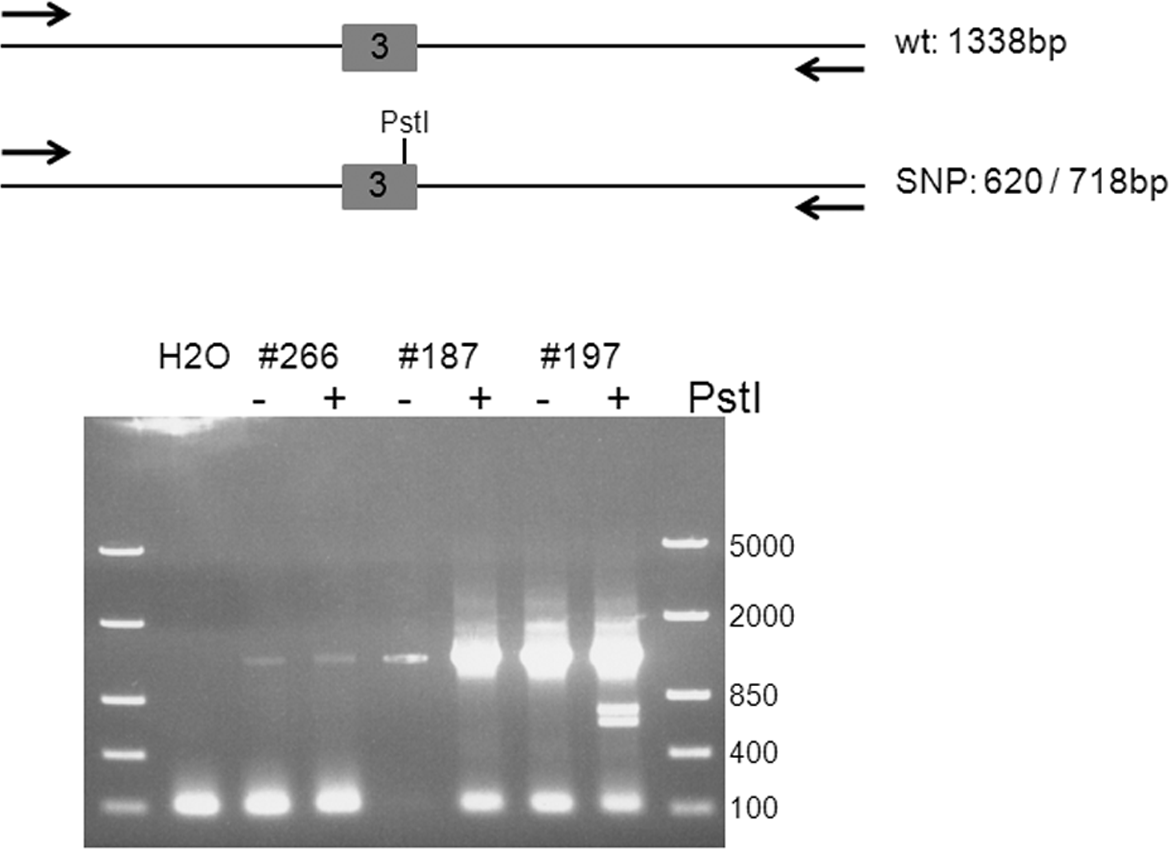

Supplement: Figure S1 — Analysis of Rb1_SNV/wt clones for presence of the SNV. Genomic DNA of three independent, correctly targeted (as judged by Southern blot) Rb1_SNVneo clones was amplified by PCR with the indicated, Rb1 exon 3 flanking, primers and the obtained product was digested with PstI. Presence of the introduced SNV results in a PstI restriction site in the amplified fragment. Digestion yields a 1338 bp fragment for the untargeted wild type allele and 718 bp and 620 bp fragments for the targeted SNV allele. Only clone 197 contains the SNV. (TIF) [file pone.0074159.s001.tif]

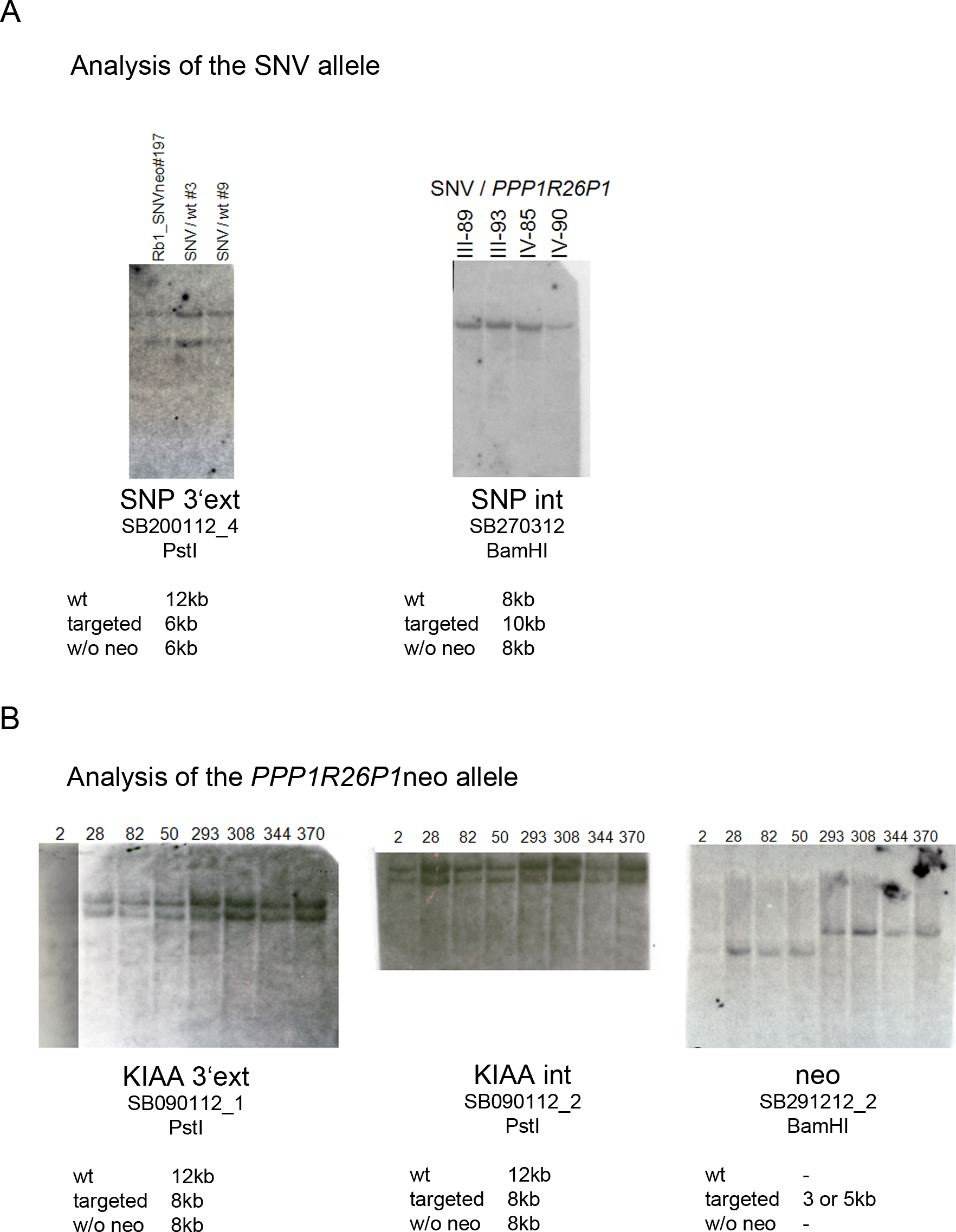

Supplement: Figure S2 — Southern blots of targeted ES cell clones. A) Analysis of the SNV allele by Southern blot. Positions of Southern blot probes are indicated in Figure 1C. left panel: genomic DNA of Rb1_SNVneo #197 and two SNV / wt clones was digested with PstI and the blot hybridized with the SNP 3’ external probe; expected bands for correctly targeted clones: 12 kb for the wild type allele, 6 kb for the targeted allele in Rb1_SNVneo and 6 kb for the targeted allele after removal of the selection cassette in SNV / wt clones. middle panel: genomic DNA of four independently targeted SNV / PPP1R26P1 clones was digested with BamHI and the blot hybridized with the SNP internal probe; expected bands for correctly targeted clones: 8 kb for the wild type allele, 10 kb for the targeted allele and 8 kb for the targeted allele after removal of the selection cassette. Occurrence of only one band indicates a single targeting event in the analyzed clones. B) Analysis of PPP1R26P1neo allele by Southern blot. Eight independently targeted SNV_PPP1R26P1neo clones were analyzed. left panel: genomic DNA digested with PstI and hybridized with the KIAA 3’ external probe is expected to result in : 12 kb for the wild type allele and 8 kb for the targeted allele. middle panel: genomic DNA digested with PstI and hybridized with the KIAA internal probe; expected bands for correctly targeted clones: 12 kb for the wild type allele and 8 kb for the targeted allele. Presence of the indicated two fragments indicates a single targeting event in the genome. right panel: genomic DNA was digested with BamHI and the blot hybridized with the neo probe recognizing the neomycin selection cassette; expected band for correct and single targeted clones: 3 or 5 kb, depending on the orientation of the selection cassette in the targeting vector. (TIF) [file pone.0074159.s002.tif]

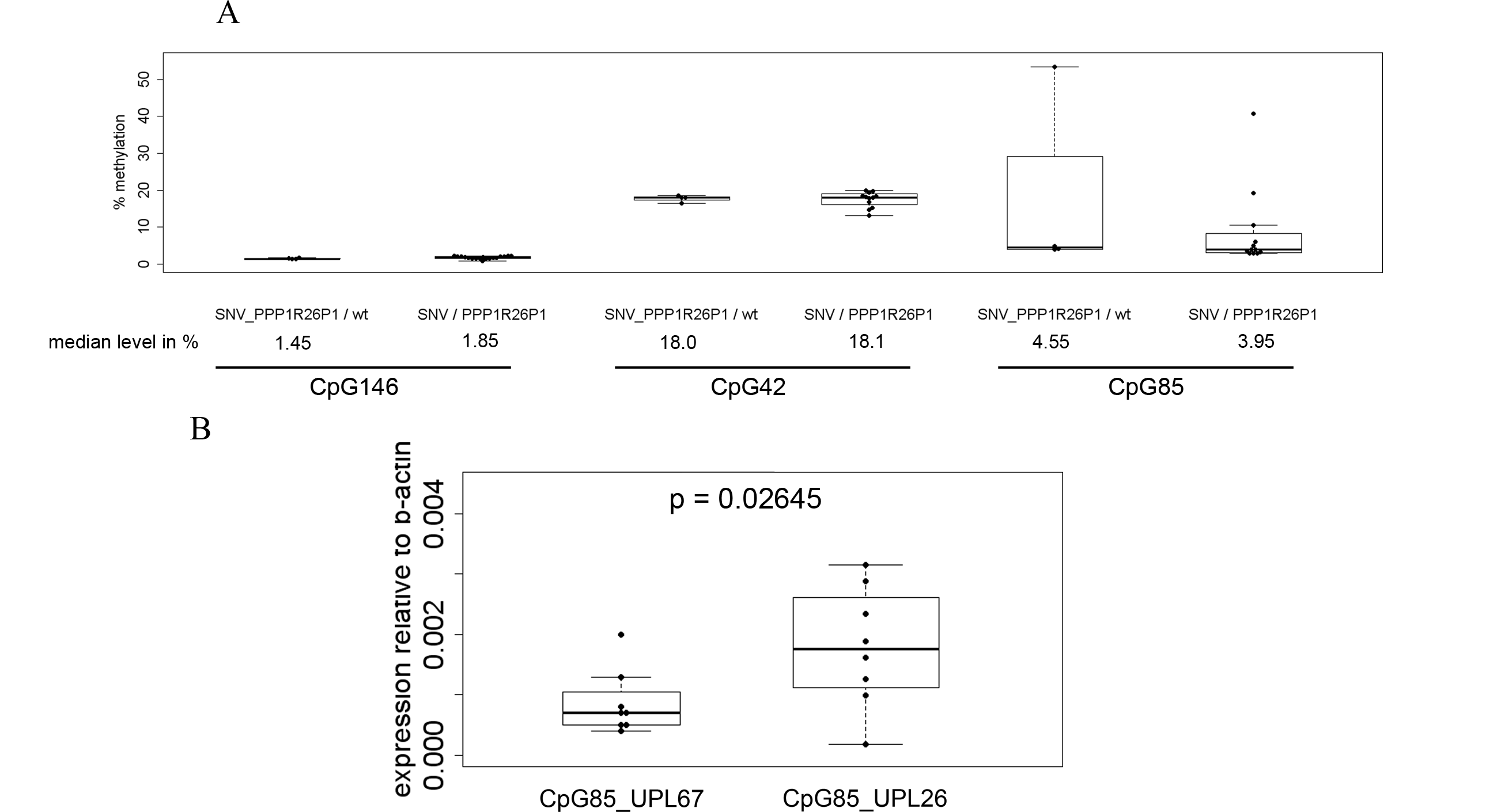

Supplement: Figure S3 — Analysis of two independent clones with genotype SNV_PPP1R26P1/wt. A) DNA methylation at CpG islands CpG146, CpG42 and CpG85. The median percentage of DNA methylation calculated over all CpG positions in all analyzed reads is indicated. Measurements for clones with genotype SNV_PPP1R26P1/wt are given on the left hand side for each CpG island, on the right hand side measurements presented in Figure 2B for genotype SNV / PPP1R26P1 are depicted for better comparability. No differences in the susceptibility to DNA methylation at all three CpG islands were observed. B) Transcript expression from CpG85. Quantitative RT-PCR using the assays located in CpG85 (as indicated in Figure 3B) show higher expression towards the 3’-end of CpG85 in cells with genotype SNV_PPP1R26P1/wt. This is in accordance to the results obtained for cells with genotype SNV / PPP1R26P1 (shown in Figure 3A). (TIF) [file pone.0074159.s003.tif]

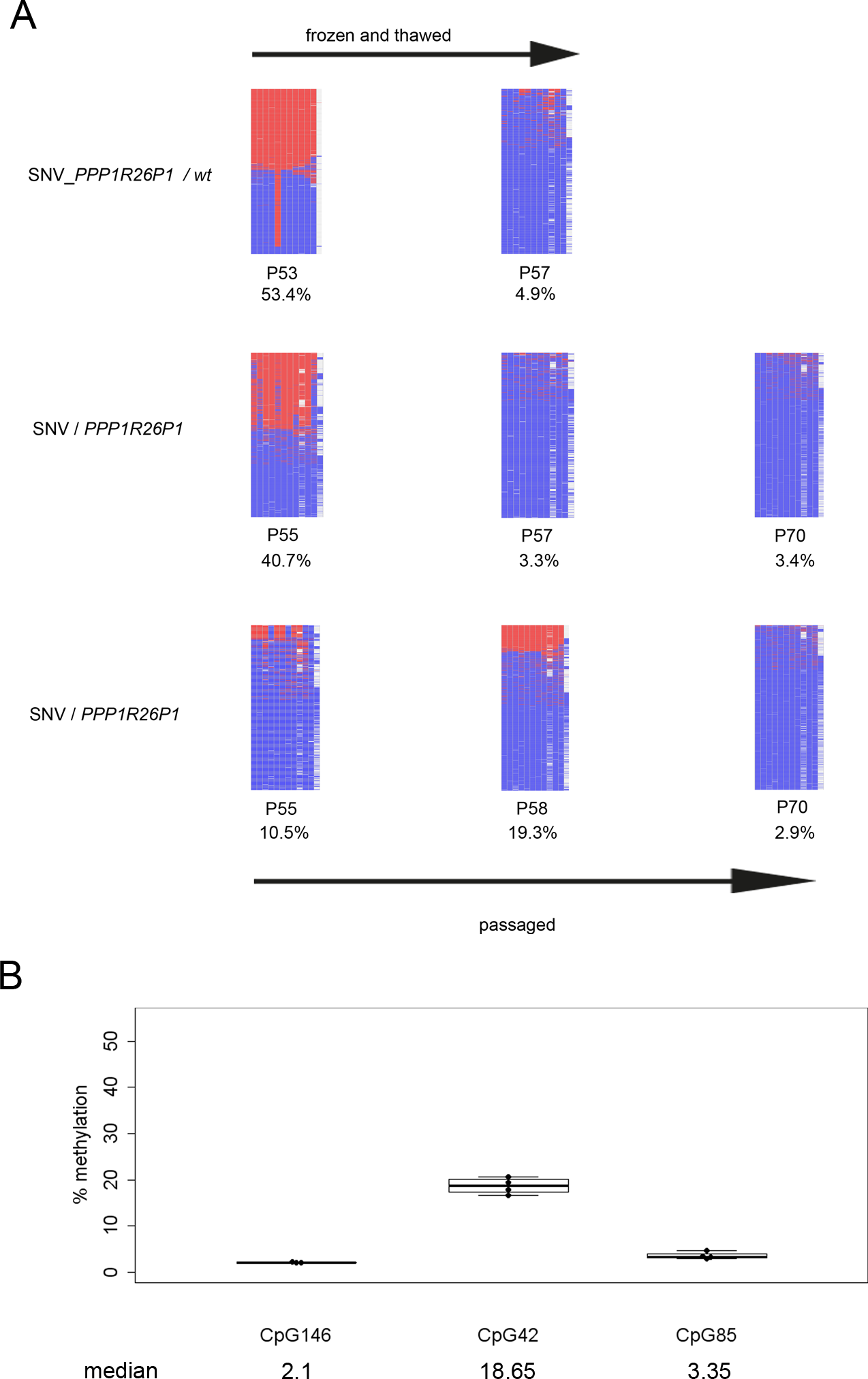

Supplement: Figure S4 — Analysis of DNA methylation in later passage cells. A) DNA methylation heat maps of next generation bisulfite sequencing of CpG85. One clone of genotype SNV_PPP1R26P1/wt and two clones of genotype SNV / PPP1R26P1 were analyzed for DNA methylation at CpG85 after one freezing and thawing (middle panel) cycle and after prolonged passaging (right panel). High transient DNA methylation at CpG85 can be observed at the initial passages (P53, P55; left panel), but it is lost after one freezing and thawing cycle (P57, P58; middle panel) and not stable during continuous culture up to passage 70 (right panel). The median percentage of DNA methylation over all CpG sites in all reads is given below the DNA methylation heat maps. Red: methylated, blue: unmethylated, white: not analyzed. B) Median levels of DNA methylation at CpG146, CpG42 and CpG85 at passage 70 measured in four independent clones with genotype SNV / PPP1R26P1. DNA methylation was analyzed by next generation bisulfite sequencing. By comparison to the levels of DNA methylation shown in Figure 2B, the median level (given below the labels) of DNA methylation remains stable over the employed 15 passages, being equivalent to about 30 cell divisions. (TIF) [file pone.0074159.s004.tif]

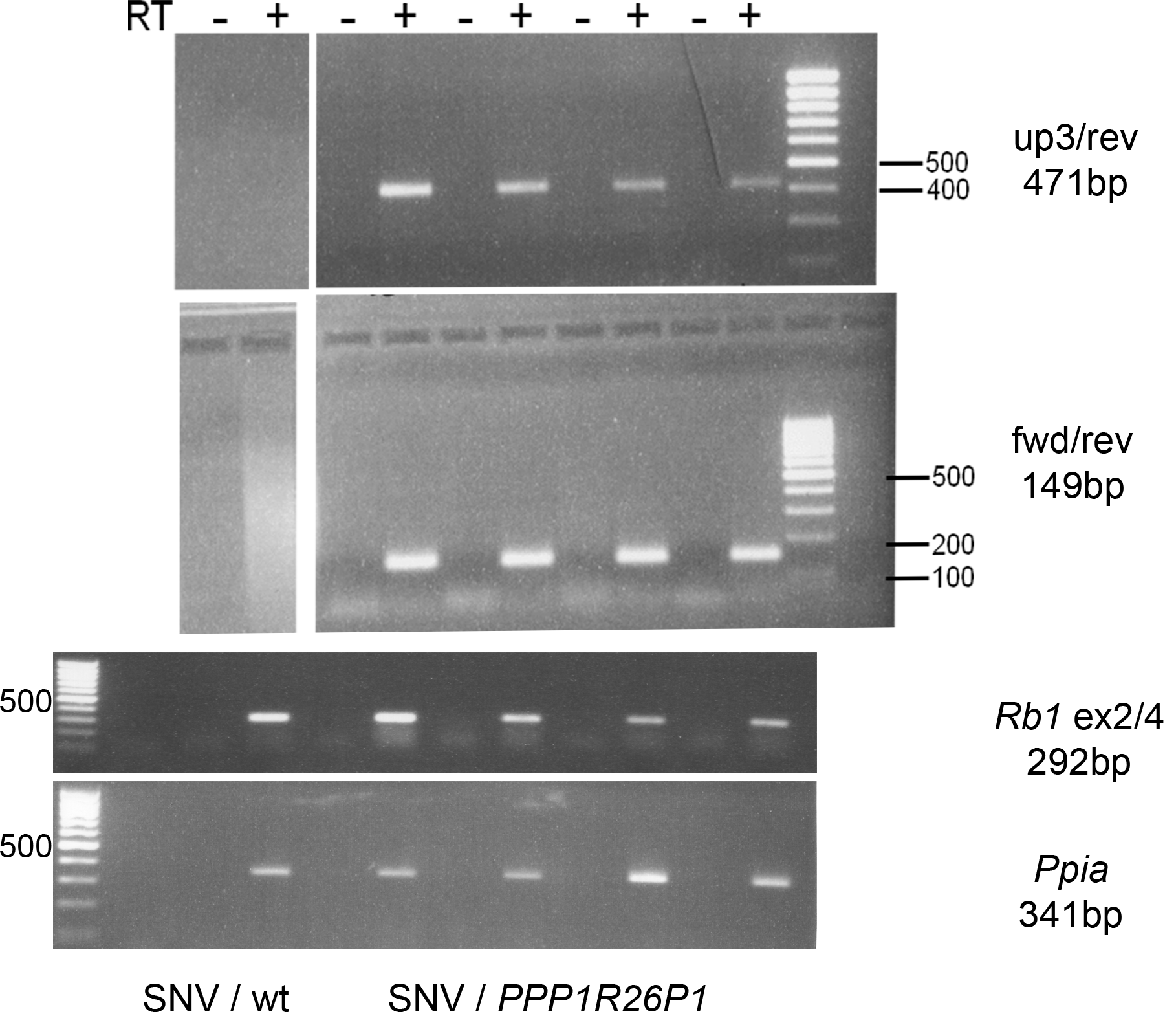

Supplement: Figure S5 — Transcript expression at CpG85. Standard RT-PCR shows expression of a transcript at the 3’-end of CpG85. Expression was detectable using two RT-PCR assays, detecting transcripts from TSSs 2 and 3 in four independent SNV / PPP1R26P1 clones but not in SNV / wt cells. Expression of Rb1 and the house keeping gene Ppia was detectable in all cell clones. (TIF) [file pone.0074159.s005.tif]
